# Protein Profiling of Blood Samples from Patients with Hereditary Leiomyomatosis and Renal Cell Cancer by Surface-Enhanced Laser Desorption/Ionization Time-of-Flight Mass Spectrometry

**DOI:** 10.3390/ijms131114518

**Published:** 2012-11-08

**Authors:** Takao Kamai, Naohisa Tomosugi, Hideyuki Abe, Yasushi Kaji, Tetsunari Oyama, Ken-Ichiro Yoshida

**Affiliations:** 1 Department of Urology, Dokkyo Medical University, Tochigi 321-0293, Japan; E-Mails: h-abe@dokkyomed.ac.jp (H.A.); yoshida@dokkyomed.ac.jp (K.-I.Y.); 2 Proteomics Research Unit, Division of Advanced Medicine, Medical Research Institute, Kanazawa Medical University, Ishikawa 920-0293, Japan; E-Mail: tomosugi@kanazawa-med.ac.jp; 3 Department of Radiology, Dokkyo Medical University, Tochigi 321-0293, Japan; E-Mail: ykaji@dokkyomed.ac.jp; 4 Department of Pathology, Dokkyo Medical University, Tochigi 321-0293, Japan; E-Mail: oyama@med.gunma-u.ac.jp

**Keywords:** Hereditary Leiomyomatosis and Renal Cell Cancer (HLRCC), fumarate hydratase (FH), surface-enhanced laser desorption/ionization time-of-flight mass spectrometry (SELDI-TOF MS), metastasis

## Abstract

Hereditary leiomyomatosis and renal cell cancer (HLRCC) is an extremely rare syndrome with autosomal dominant inheritance. HLRCC is characterized by a predisposition to leiomyomas of the skin and the uterus as well as renal cell carcinoma. The disease-related gene has been identified as fumarate hydratase (fumarase, FH), which encodes an enzyme involved in the mitochondrial tricarboxylic acid cycle. Protein profiling may give some insight into the molecular pathways of HLRCC. Therefore, we performed protein profiling of blood samples from HLRCC patients, their family members, and healthy volunteers, using surface-enhanced laser desorption/ionization time-of-flight mass spectrometry (SELDI-TOF MS) coupled with IMAC-Cu chips. For hierarchical clustering analysis, we used the 45 peaks that revealed significant differences in single-marker analysis over the range from 1500 to 15,000 *m*/*z*. Heat map analysis based on the results of clustering distinguished the HLRCC kindred from non-HLRCC subjects with a sensitivity of 94% and a specificity of 90%. SELDI-TOF MS profiling of blood samples can be applied to identify patients with HLRCC and to assess specific molecular mechanisms involved in this condition.

## 1. Introduction

Hereditary leiomyomatosis and renal cell cancer (HLRCC, Online Mendelian Inheritance in Man accession number 605839) is a recently identified autosomal dominant tumor susceptibility syndrome characterized by a predisposition for the development of benign leiomyomas of the skin and the uterus (fibroids and myomas), as well as aggressive renal cell carcinoma with papillary type 2 or collecting duct histology [1–4]. The disease-related gene has been identified as fumarate hydratase (fumarase, FH, Online Mendelian Inheritance in Man accession number 136850) located at 1q43 [1]. FH encodes an enzyme that is part of the mitochondrial tricarboxylic acid (Krebs) cycle involved in cellular energy metabolism and appears to function as a tumor suppressor since its activity is very low or absent in tumors from individuals with HLRCC [1–6]. In addition, loss of heterozygosity (LOH) has been found in the tumors of affected individuals, resembling a classic Knudson “two-hit” model [1–5]. In HLRCC, there is a germline mutation and subsequent somatic loss of the second allele results in tumorigenesis. However, it appears that other genetic or environmental factors might be involved in causing an increase of susceptibility to renal cancer in addition to FH mutation [6].

There is evidence that early detection of various forms of cancer can lead to an improved clinical outcome [7]. In addition to classic serum biomarkers, other techniques such as imaging, cytology, and serology can also play a major role in early diagnosis or in the identification of precancerous lesions. Accordingly, there is an urgent need to discover and validate novel biomarkers or other diagnostic modalities. How can new biomarkers be discovered? Sequencing of the human genome has provided us with a list of all human genes, and this could lead to the development of specific reagents that will allow testing of thousands of proteins as potential biomarkers for human diseases. One of the methods used for cancer biomarker discovery is the candidate protein approach, in which a particular protein is tested in samples from normal individuals and patients with cancer to determine its discriminatory value. However, comparative multiparametric analysis of serum samples by quantitative mass spectrometry to differentiate healthy and disease states may be more useful. To date, despite extensive investigations employing these technologies, no major cancer biomarkers have been discovered or validated. Mass spectrometry currently represents the most important analytical tool for proteomic studies [8]. This method can identify proteins and peptides with relative ease and allows multiparametric analysis of complex biological fluids such as serum. In the present study, we examined serum samples from a Japanese HLRCC kindred by surface-enhanced laser desorption/ionization time-of-flight mass spectrometry (SELDI-TOF MS) and attempted to discriminate them from samples provided by healthy controls. We found that quantitative mass spectrometry could be applied to identify patients with HLRCC. To our knowledge, this is the first report about mass spectrometry profiling of blood samples from HLRCC kindred. It was hoped that the information obtained might contribute to elucidation of the molecular mechanism and promised establishment of biomarkers in HLRCC in the forthcoming study.

## 2. Case Presentation

### 2.1. Pedigree 1

**Figure 1:**
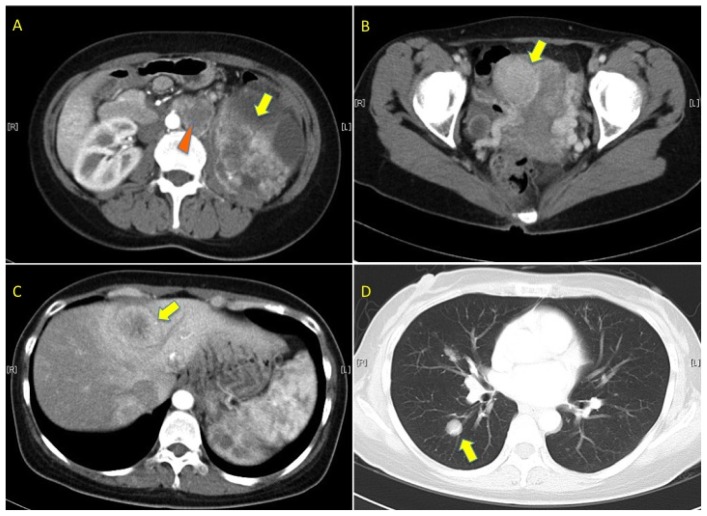
Computed tomography (CT) imagings of the proband of pedigree 1. (**A**) Enhanced CT scan of the patient’s abdomen showing a large enhanced and some necrotic left renal tumors (arrow). There are also multiple enlarged para-aortic lymph nodes (arrowhead); (**B**) Enhanced pelvic CT demonstrates multiple large uterine leiomyomas (arrow); (**C**) Enhanced abdominal CT shows multiple liver lesions (arrow); (**D**) Chest CT scan shows multiple lung lesions (arrow). The liver lesion shown by the arrow in **C** and the lung lesion shown by the arrow in (**D**) are examples of multiple lesions.

**Figure 2:**
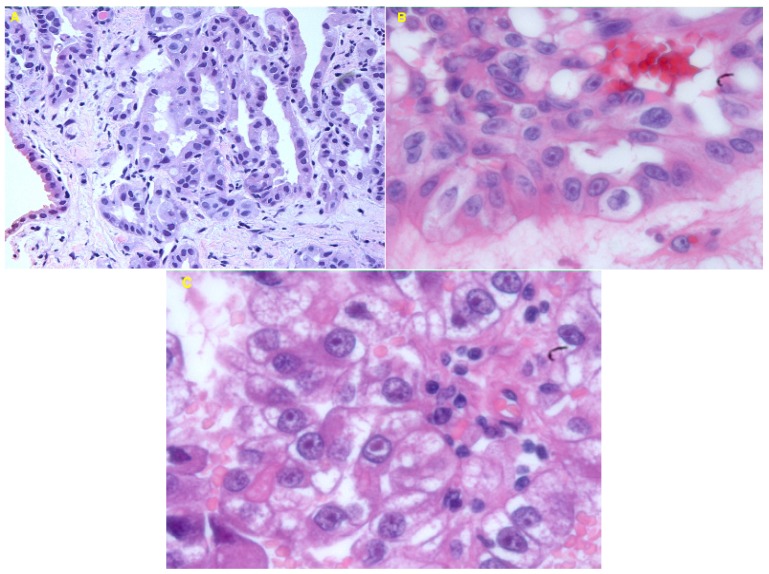
Microscopic features of three examples of hereditary leiomyomatosis and renal cell cancer (HLRCC). (**A**) Proband (III-9) from pedigree 1. Fine needle aspiration of the renal tumor revealed a tubulo-papillary architecture with prominent nucleoli (hematoxylin and eosin, 20×); (**B**) III-4 from pedigree 1. The renal cancer cells show prominent eosinophilic nucleoli, some surrounded by a clear halo (hematoxylin and eosin, 100×); (**C**) The female from pedigree 2. The renal tumor consists of cells with eosinophilic cytoplasm and large nucleoli surrounded by clear halo-like spaces (hematoxylin and eosin, 100×).

A 39-year-old Japanese woman (case III-9 in pedigree 1) was presented to the urological clinic of our hospital in May 2007. She complained of intermittent gross hematuria and a progressively enlarging painless left flank mass for three months. She had no significant past medical or surgical history. Imaging studies revealed a large cystic and solid left renal tumor with a diameter of 17 cm, para-aortic lymph node tumors, multiple lung and liver tumors, and a uterine leiomyoma with a diameter of 7 cm (Figure 1). Laboratory tests revealed mild anemia (Hb 9.7 g/dL) and elevation of serum C-reactive protein (CRP). Needle biopsy of the renal and liver tumors demonstrated carcinoma with a tubulo-papillary architecture with a large nucleus (Figure 2A). Given these findings, primary renal carcinoma was suspected.

**Figure 3:**
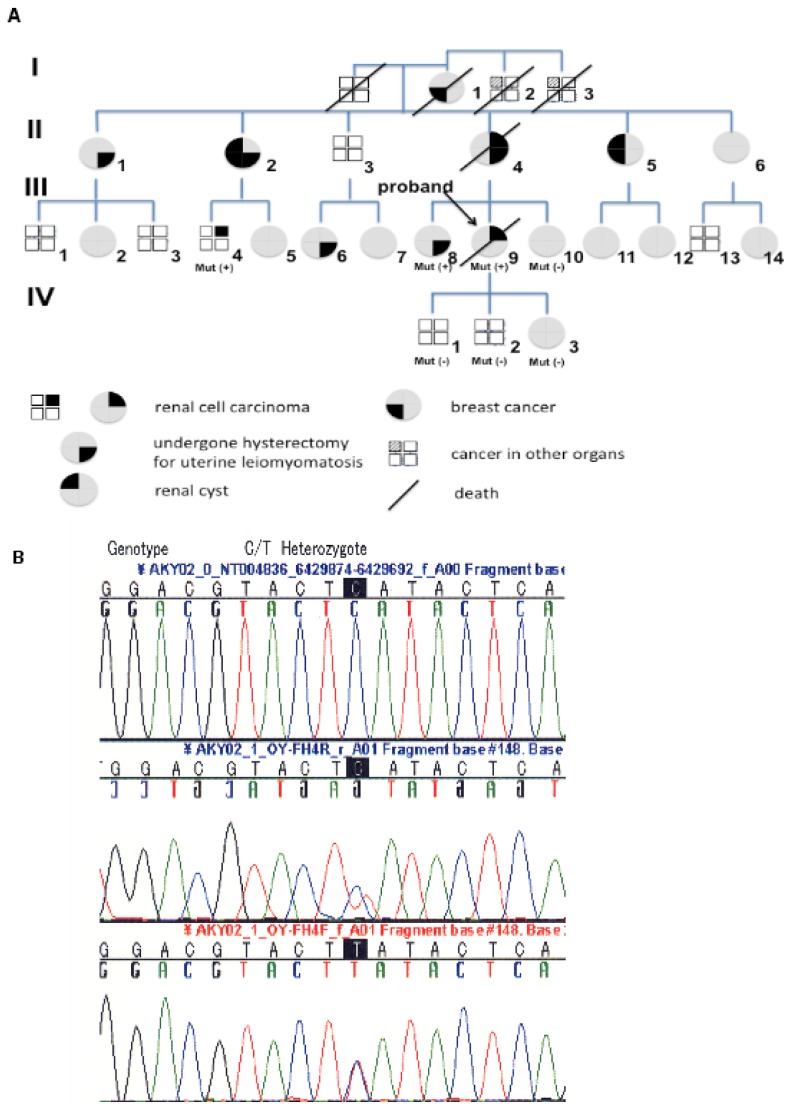
Pedigree and mutation analysis. (**A**) Pedigree of family 1. Generations are represented by Roman numerals and individuals are shown by Arabic numerals. The index patient (proband) is III-9, and is indicated by the arrow. “Mut” shows mutation screening. “Mut +” and “Mut −” indicate mutation-positive and mutation-negative individuals, respectively; (**B**) FH mutations in the patients. Sequencing chromatograms of genomic DNA from a control subject and patients III-4, -8, and -9. Sequence analysis revealed a germline C to T mutation at cDNA position 574 (C574T) that changes histidine to tyrosine at codon 192 (H192Y), suggesting loss of the wild-type FH allele.

The family history revealed that the proband’s mother (case II-4 in pedigree 1) had been diagnosed with left renal collecting duct carcinoma and multiple bone metastasis at the age of 58 years and died within six months. The patient’s maternal cousin (a 42-year-old man, case III-4 in pedigree 1) had undergone radical left nephrectomy for renal tumors, and had since remained recurrence-free for four years. Pathological examination of his surgical specimen revealed that tumor cells each had a large nucleus with an eosinophilic nucleolus that was surrounded by a clear halo (Figure 2B). Furthermore, five female relatives, including the patient’s mother, the patient’s sister, her maternal aunt and her cousin, had undergone hysterectomy for uterine leiomyomatosis (Figure 3A). From this family history, familial renal cancer was suspected. However, there was no clinical or imaging evidence of von Hippel-Lindau (VHL) disease or hereditary papillary renal cancer (HPRC) syndrome [9].

We performed FH mutation screening by sequencing genomic DNA with the polymerase chain reaction (PCR) in seven maternal family members according to the method described previously [10]. This study was conducted in accordance with the Helsinki Declaration and institutional review board approval was obtained. The patient signed a consent form approved by the Committee on Human Rights in Research at our institution. A novel FH mutation (C574T) was detected in three members (III-9; the proband, III-8; proband’s sister, III-4; proband’s cousin) (Figure 3B). The codon involved was H192Y and this mutation has not been described, previously.

The patient received best supportive care for her disease and died in January 2009.

### 2.2. Pedigree 2

After we encountered the above-mentioned Japanese HLRCC kindred, another female patient was identified in our records. A 26-year-old Japanese woman had received right radical nephrectomy for renal tumors in September 2001. Histological examination revealed papillary type 2 renal cell carcinoma, with moderate differentiation (pT1bpN0M0). The tumor cells each had a large nucleus with an eosinophilic nucleolus that was surrounded by a clear halo (Figure 2C). Her mother and sister had undergone hysterectomy for uterine leiomyomatosis. The patient has remained alive and free of recurrence for 11 years since her surgery. It seemed possible that this represents another HLRCC kindred based on the clinical features and family history. When asked to undergo mutational analysis, the patient and her family did not give approval.

## 3. Results

### 3.1. Histological Examination

Three renal cell cancers (case III-4 and III-9 in pedigree 1, the female in pedigree 2), were composed of cells with very prominent nuclei and large nucleoli (Figure 2A–C). In particular, the hallmark of these tumors was an orangiophilic or eosinophilic nucleolus with a perinucleolar halo reminiscent of cytomegalovirus infection (hematoxylin and eosin, 100×) (Figure 2B,C).

### 3.2. Mutation Screening

A novel FH mutation (C574T) was detected in three patients from kindred 1 by direct sequencing of the *FH* gene from leukocyte DNA (Figure 3B). The mutation was located on codon H192Y and it has not been described before.

The female renal cancer patient from pedigree 2 did not agree to undergo mutation analysis.

### 3.3. SELDI-TOF MS

**Figure 4:**
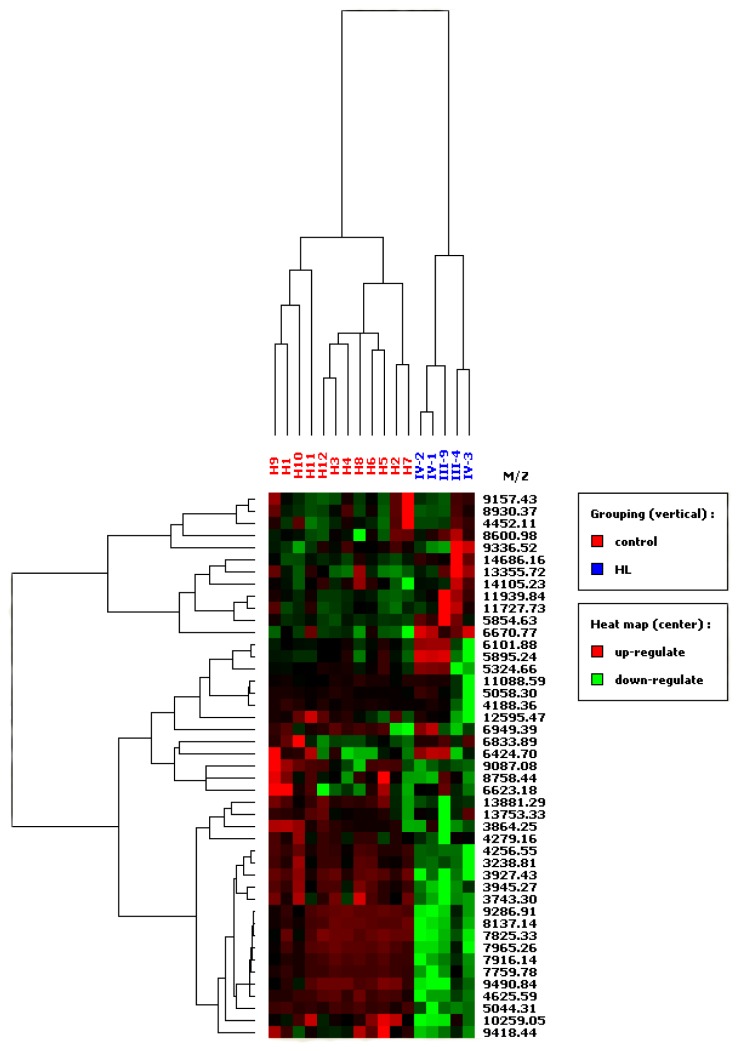
Heat map based on the results of protein profiling and hierarchical clustering analysis. The horizontal line above the heat map represents the case number (blue: HLRCC family member, red: healthy volunteer controls). Patients designated as III-4, III-9, and IV-1 to IV-3 belong to pedigree 1. Vertical lines represent the 45 significant peaks.

**Figure 5:**
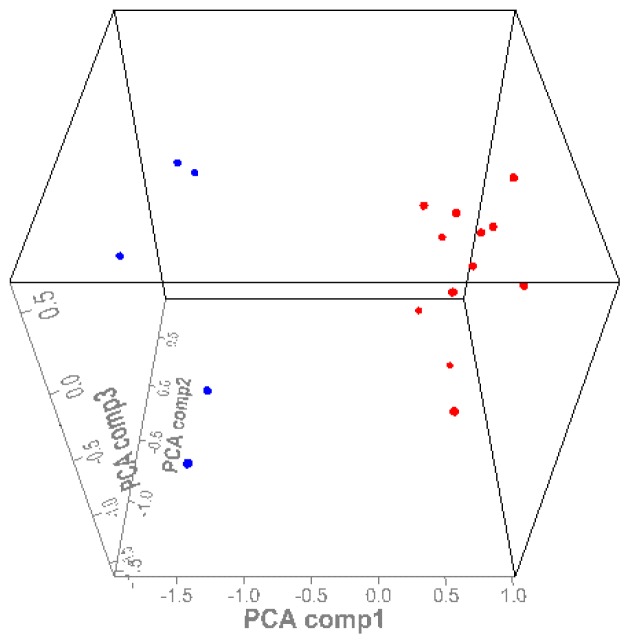
Three-dimensional principal component analysis (PCA) of protein profiling. Cluster A (blue: HLRCC), cluster B (red: healthy volunteers). Samples from the two groups are highly concentrated in certain areas.

Protein peaks were selected by employing a first pass signal-to noise ratio of 5, followed by a second pass signal-to-noise ratio of 2 and peak selection at 0.3% of the mass window. After preliminary analysis of protein spectra, the selected protein peaks were subjected to HCA analysis. To create a heat map, the 45 significant peaks identified by individual analysis were all used. On the resulting heat map (Figure 4), patients who had familial renal carcinoma were discriminated from controls. Principle component analysis (PCA) was performed to assess the differences of proteins between the two groups, revealing separation between the HLRCC group and the control group (Figure 5).

In HLRCC kindred samples, single-marker analysis detected 4 peaks that were significantly larger and 21 peaks that were significantly smaller compared with the peaks in serum from the controls. Data on the significant peaks detected by single-marker analysis (*p* < 0.05) are shown in Table 1.

**Figure 6:**
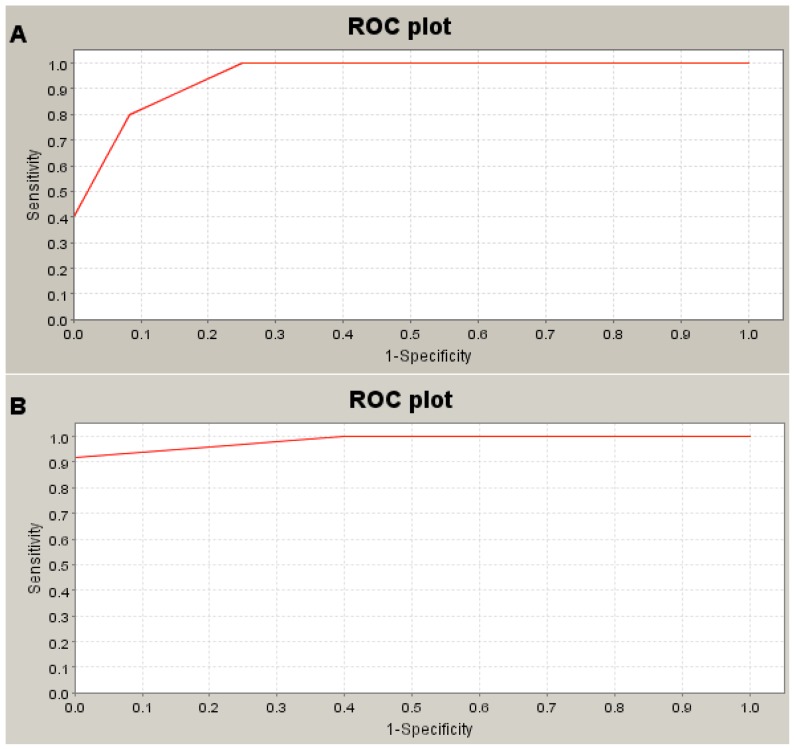
Representative receiver operating characteristic curves (ROC) for the detection of HLRCC. (**A**) When the curve was plotted for the enlarged peak at 5855 *m*/*z* (**A**), the area under the curve (AUC) was 0.95. (**B**) For the reduced peak at 3239 *m*/*z*, AUC was 0.98.

To determine the sensitivity and specificity of potential serum protein biomarkers and the usefulness of quantifying protein peaks for identification of HLRCC, receiver operating characteristics (ROC) analysis was conducted. The area under the ROC curve was 0.95 for each of three larger peaks (*m*/*z* 5855, 6671, and 14,686), showing a sensitivity of 90% and a specificity of 82% (Figure 6A). The ROC area for a representative smaller peak (3239 *m*/*z*) was 0.98, showing a sensitivity of 94% and a specificity of 90% (Figure 6B). However, we could not determine the 95% confidence intervals (CI) for the sensitivity and specificity using the attached software. Therefore, we analyzed the mean ± SD in healthy persons and in HLRCC pedigree 1, and we have summarized the data in Table 1. The data suggested that quantification of these peaks by SELDI-TOF/MS was a useful diagnostic tool for HLRCC.

## 4. Discussion

Many lines of evidence have indicated a strong genetic component to hereditary leiomyomatosis and renal cell cancer (HLRCC), and mutation of fumarate hydratase (FH) at 1q43 has been found to cause this condition [1]. A number of studies have demonstrated that papillary type 2 or collecting duct carcinoma can be an aggressive form of inherited renal cell cancer [2–4,11]. Available reports support pseudo-hypoxia as the mechanism of tumorigenesis in HLRCC [12,13]. Fumarate accumulates in HLRCC patients due to loss of FH activity, then competitively inhibits the hypoxia-inducible factor (HIF) prolyl hydroxylase. As a result, HIF remains unhydroxylated and avoids degradation, thus upregulating the transcription of several genes involved in angiogenesis and cell proliferation, including vascular endothelial growth factor (VEGF) and platelet-derived growth factor (PDGF) [12–15]. As was seen in the proband’s cousin (III-4), who was followed up for a renal cyst, such cystic change may represent an early stage in carcinogenesis [16]. In a mouse model, Pollard *et al.* showed that inactivation of Fh1 in the kidney causes activation of HIF and increases cell proliferation to produce renal cysts derived from the collecting ducts and the thick ascending limb of the loop of Henle [17]. It has also been reported that the collecting duct may be the site of origin of some renal carcinomas in HLRCC [4,11]. On the other hand, Adam *et al.* reported that renal cyst formation in Fh1-deficient mice is independent of the HIF pathway [18]. These findings not only suggest that renal cysts attributable to reduced FH activity may be responsible for some sporadic renal cancers, so patients with such cysts need active surveillance, but also that the pseudo-hypoxia hypothesis of carcinogenesis in FH patients does not fully explain FH-associated tumorigenesis.

FH is an enzyme that exists in both the mitochondria and the cytoplasm of all eukaryotes. It is involved in generating energy through a metabolic pathway of the Krebs cycle in the mitochondria, but the role of FH in the cytoplasm is unclear. In addition to competitive inhibition of members of the 2-oxoglutarate-dependent oxygenase superfamily, including the histone demethylase enzymes (HDMs) and HIF prolyl hydroxylase, there is a growing body of evidence to show that loss of FH activity and the resulting increase of fumarate has multiple inter-related consequences, including abnormal metabolism possibly linked to reductive carboxylation, mitochondrial dysfunction, an abnormal response to DNA damage, and dysregulation of nuclear factor (erythroid-drived 2)-like 2 (Nrf2) signaling. Such findings suggest that FH-associated diseases might develop in an HIF-independent manner [18–24]. Accordingly, the identification of loss- or gain-of-function mutations of key metabolic enzymes, including FH, may shed light on the role of altered metabolism in FH-associated tumorigenesis [23].

It is likely that HLRCC features a single germline mutation of FH, with subsequent somatic loss of the second allele resulting in tumorigenesis. So far, FH mutations have been identified in 37 of 46 (80%) kindreds from the UK and all three Finnish kindreds examined [3,5] as well as in 31 out of 35 (89%) North American kindreds [7]. Thus, 76 out of a total of 89 kindreds (85%) analyzed worldwide have been found to possess the FH mutation. This suggests the possibility of genetic heterogeneity. IN fact, four UK probands investigated all had decreased FH activity without an identifiable FH mutation [3]. The FH gene alterations detected so far have all led to loss of enzyme activity and have included missense, frameshift, and nonsense mutations, as well as whole gene deletion, but no genotype-phenotype correlations have yet been observed [25]. Thus, genetic testing is available for the diagnosis of HLRCC, but not all patients have FH mutations, suggesting that other causative genes or environmental factors may still be discovered.

Mass spectrometry has been used in two different settings in the field of cancer diagnostics. First, to discover novel cancer biomarkers, biological fluids (serum, urine, cerebrospinal fluid, *etc.*) are fractionated by chromatographic techniques and analyzed by mass spectrometry to identify new protein markers. Second, mass spectrometry is used to generate a profile of peaks from serum after chromatography (a protein chip) has been employed for the immobilization of certain proteins or peptides. Petricoin *et al.* employed powerful bioinformatic algorithms to discriminate between health and disease states with unprecedented sensitivity and specificity, without knowing the identity of these peaks [26]. This approach has already been used for the diagnosis of several cancers [27].

SELDI-TOF MS has been widely applied for examination of serum and plasma in attempts to discover proteomic changes that are useful for the diagnosis of human cancers [28–32]. This method employs on-chip retentate chromatography followed by matrix assisted laser desorption/ionization (MALDI) time-of-flight mass spectrometry (MALDI-TOF MS) to generate spectra or “proteomic profiles” of biological fluids. The SELDI ‘ProteinChip Arrays’ used for profiling studies are typically immobilized metal ion (IMAC) or ion exchange surfaces. Tumor-related proteolytic activity might produce disease-specific patterns of small proteolytic fragments (<15 kDa) that could be detectable by mass spectrometry. If these findings obtained by SELDI-TOF/MS can be reproduced and validated, it could represent a major breakthrough with immediate clinical applicability.

Although we reported on the first Japanese HLRCC kindred, our study had a small sample size, so several issues have not yet been elucidated. First, although the relevant proteins were not identified in the present study, the higher and lower protein peaks distinguished the HLRCC kindred from non-HLRCC subjects with a sensitivity of 94% and a specificity of 90%. This finding suggested that the HLRCC subject from pedigree 1 possesses a distinct proteomic signature from the other subjects, but it has not been proven that this subject’s HLRCC is caused by an FH mutation. This hereditary cancer syndrome is novel, so additional causative genes may still be discovered. Furthermore, our method did not allow calculation of the 95% CI for the sensitivity and specificity by using the attached software. This might have been a particular problem given the small sample size because of the rarity of HLRCC. Second, although the female patient in pedigree 2 did not agree to undergo mutation analysis, histologic examination of her tumor revealed that the cells had eosinophilic nucleoli with a perinucleolar halo (reminiscent of renal cancer in HLRCC) [11]. By combining this female from pedigree 2 together with the above-mentioned five members of pedigree 1, heat map analysis revealed that these six subjects could be classified into one group, and distinguished them from non-HLRCC subjects with a sensitivity of 90% and a specificity of 85% (data not shown). These findings indicate that HLRCC families might have a distinct proteomic profile from non-HLRCC subjects, or else that pedigree 2 is (distantly) related to pedigree 1. The latter possibility could be confirmed if the same mutation was found as shared by pedigree 2 and pedigree 1. Third, although the children of the proband in pedigree 1 (two sons and one daughter; IV-1 to IV-3) showed no mutations of FH, the heat map assigned them to this HLRCC family and not to the non-cancerous control group, suggesting that we may not be discriminating HLRCC patients (III-4, III-9) from normal persons (IV-1 to IV-3), but may be separating families from each other. We need to explore the specific proteins that are related to the difference between the two groups, which could help to explain why we were unable to differentiate unaffected from affected relatives. Our study was only exploratory, and we intend to apply our method to a larger sample in order to perform in-depth analysis of the peptidome and proteome to fully assess the diagnostic potential of the changes detected in a future study.

At present, we cannot determine whether the peaks revealed by quantitative mass spectrometry represent novel biomarkers or are simply abundant nonspecific proteins. However, mutation screening by polymerase chain reaction (PCR) sequencing of genomic DNA and biological mass spectrometry combined with bioinformatic analysis may be useful tools to elucidate the molecular basis of various diseases, which may eventually yield clinically useful diagnostic and surveillance methods.

## 5. Materials and Methods

### 5.1. Mutation Screening

We collected serum samples from seven maternal family members in kindred 1 (Figure 3A). We screened for mutations by using genomic DNA and PCR primers designed to amplify known or predicted exons and the flanking intronic sequence. For single-stranded conformational polymorphism (SSCP) analysis, samples that showed bandshifts were re-amplified by PCR and sequenced. Doragon Genomics Center (Takara Bio Ltd., Mie, Japan) performed these analyses according to the reported methods [10]. Briefly, a sample of whole blood (10 mL) was collected from each patient and was diluted in PBS with 2 mM EDTA. Then peripheral blood mononuclear cells (PBMC) were separated by using gradient centrifugation and Ficol-Hypaque medium (Biocoll, Berlin, Germany). Total RNA was isolated with an RNeasy kit (Qiagen, Hamburg, Germany), including a DNA digestion step using the RNase-free DNase set. The primers used for exons 0 to 9 of FH have been reported previously [10]. Real-time RT-PCR was performed in a 20 μL reaction mixture (containing 10 ng of sample cDNA, 5.0 pmol sense primer, 5.0 pmol anti-sense primer, 2.0 μL of 10× Ex Taq buffer, 2.0 μL of dNTP mixture, and 0.2 μL of Ex Taq), with 35 cycles of 94 °C for 30 s, 58 °C for 30 s, and 72 °C for 45 s. To carry out nucleotide sequencing, purified PCR products were used as the template for cDNA synthesis and the above-mentioned exon primers were also used.

### 5.2. Serum Protein Profiling by SELDI-TOF/MS

We collected serum samples from five members of kindred 1, as well as from 12 healthy controls. Samples were adjusted to an equal protein concentration. We performed these analyses according to the reported methods [33]. Briefly, after trials of surface-enhanced laser desorption/ionization time-of-flight mass spectrometry (SELDI-TOF MS) methods using a pooled sample, the samples were assayed by SELDI (with/without de-salting) and Cu^2+^ loaded immobilized metal affinity capture (IMAC) ProteinChip Arrays (IMAC30; Bio-Rad, Fremont, CA, USA). SELDI-TOF MS was performed with an IMAC 30 because of the good reproducibility of this method for detecting proteins in serum. In brief, 40 μL of diluted serum was applied to an IMAC 30 chip array and incubated at room temperature for 30 min. The chip array was washed and air-dried. Then 0.5 μL of a-cyano-4-hydroxy-cinnamic acid (CHCA) was added twice to the array surface, and the array was analyzed with the ProteinChip System Series 4000 (Bio-Rad). The mass–charge ratio (*m*/*z*) of each of the proteins/peptides captured on the array surface was determined relative to the following external calibration standards: Arg8-vasopressin (1084.25 Da), somatostatin (1637.9 Da), dynorphin (2147.5 Da), bovine insulin b-chain (3495.95 Da), human insulin (5807.65 Da), bovine ubiquitin (8564.8 Da), and bovine cytochrome C (12,230.9 Da). The peak intensities were normalized for the total ion current by using Ciphergen Express Data Manager software, version 3.0 (Bio-Rad: Fremont, CA, USA, 2006) to compensate for variations in the amount of sample loaded onto each spot.

### 5.3. Statistical Analysis

We determined the area under the receiver operating characteristic (ROC) curve to assess the diagnostic value of protein peaks measured by SELDI TOF/MS, either individually or in combination, as previously reported [33]. For exploratory cluster analysis, principle component analysis (PCA) of the preprocessed data was done. We performed all statistical analyses with SPSS 16.0J software (SPSS, Chicago, IL, USA, 2007). For the 45 peaks that revealed significant differences in the individual analyses, hierarchical cluster analysis (HCA) was subsequently performed to create a heat map using Ciphergen Express Data Manager software, version 3.0 (Bio-Rad), according to the reported methods [33].

## 6. Conclusion

In this study, a heat map on mass spectrometry data was able to discriminate the HLRCC kindred from non-HLRCC subjects with a sensitivity of 94% and a specificity of 90%. SELDI-TOF MS profiling of blood samples can be applied to identify patients with HLRCC.

## Figures and Tables

**Table 1:** Statistical data of significant peaks detected in single-marker analysis.

Peaks in HLRCC	*m*/*z*	Healthy control (*n* = 2)		HLRCC of pedigree 1 (*n* = 5)		*p* value	ROC area
	
		mean ± SD	95% CI	mean ± SD	95% CI		
up-regulated	5,855	4.277 ± 0.520	0.330	8.517 ± 4.300	5.339	0.004	0.95
	6,671	50623 ± 1.266	0.805	9.637 ± 1.761	2.187	0.003	0.95
	14,484	1.603 ± 0.222	0.141	2.714 ± 1.219	1.514	0.003	0.95
	11,940	1.840 ± 0.314	0.199	3.396 ± 1.679	2.084	0.011	0.85

down-regulated	3239	6.589 ± 4.529	2.877	0.843 ± 0.434	0.538	0.002	0.98
	3,743	3.785 ± 2.778	1.765	0.429 ± 0.184	0.229	0.002	0.98
	3,927	37.245 ± 26.749	16.996	0.175 ± 0.058	0.177	0.002	0.98
	3,945	15.475 ± 11.430	7.262	0.847 ± 0.641	0.859	0.002	0.98
	4,257	21.837 ± 12.996	8.257	0.297 ±0.320	0.398	0.002	0.98
	4,626	18.029 ± 3.370	2.141	3.620 ± 1.908	2.369	0.002	0.98
	5,044	20.584 ± 5.844	3.713	0.360 ± 0.338	0.419	0.002	0.98
	7,760	85.036 ± 21.119	13.418	6.305 ± 10.962	13.611	0.002	0.98
	7,825	4.868 ± 1.212	0.771	0.717 ± 0.432	0.536	0.002	0.98
	7,916	4.699 ±1.146	0.728	0.621 ± 0.511	0.635	0.002	0.98
	7,965	7.789 ± 1.861	1.182	1.002 ± 1.138	1.414	0.002	0.98
	8,137	17.275 ± 4.321	2.745	2.071 ± 1.848	2.295	0.002	0.98
	9,287	74.914 ± 17.311	10.999	12.774 ± 10.948	13.594	0.002	0.98
	9,491	10.193 ± 1.988	1.263	3.543 ± 1.764	2.190	0.002	0.98
	9,418	7.693 ± 2.733	1.762	4.088 ± 0.898	1.116	0.003	0.93
	13,881	5.568 ± 1.154	0.733	3.653 ± 0.873	1.085	0.006	0.93
	4,188	7.414 ± 2.517	1.599	3.472 ± 3.244	4.028	0.020	0.88
	9,087	9.829 ± 4.014	2.550	5.559 ± 0.827	1.027	0.015	0.88
	10,259	5.537 ± 3.738	2.375	1.370 ± 1.296	1.610	0.011	0.88
	3,864	11.781 ± 3.558	2.261	6.636 ± 2.746	3.410	0.035	0.83
	4,279	7.455 ± 4.752	3.019	2.944 ± 1.901	2.360	0.020	0.83
	13,881	5.568 ± 1.154	0.733	3.653 ± 0.873	1.085	0.006	0.93
	4,188	7.414 ± 2.517	1.599	3.472 ± 3.244	4.028	0.020	0.88
	9,087	9.829 ± 4.014	2.550	5.559 ± 0.827	1.027	0.015	0.88
	10,259	5.537 ± 3.738	2.375	1.370 ± 1.296	1.610	0.011	0.88
	3,864	11.781 ± 3.558	2.261	6.636 ± 2.746	3.410	0.035	0.83
	4,279	7.455 ± 4.752	3.019	2.944 ± 1.901	2.360	0.020	0.83

## Abbreviations

HLRCC: Hereditary Leiomyomatosis and Renal Cell Cancer
FH: fumarate hydratase
